# Rifampicin as an antivirulence adjunct in hypervirulent/hypermucoviscous *Klebsiella pneumoniae* infections: a scoping review

**DOI:** 10.1186/s12879-026-13723-7

**Published:** 2026-06-05

**Authors:** Danavath Nagendra, Asha K. Rajan, Thejesh Srinivas, Vandana K.E, Aruna Poojary, Abduallah Zaawari, Gagana Hanumaiah, Neeraja R, Ancita Lobo, Souvik Chaudhuri

**Affiliations:** 1 Department of Critical Care, Kasturba Medical College, Manipal Academy of Higher Education, Manipal, Karnataka 576104 India; 2 Department of Pharmacy Practice, Manipal College of Pharmaceutical Science, Manipal Academy of Higher Education, Manipal, Karnataka 576104 India; 3 Department of Microbiology, Kasturba Medical College, Manipal Academy of Higher Education, Manipal, Karnataka 576104 India; 4 Department of Microbiology, Breach Candy Hospital Trust, Mumbai, Maharashtra 400026 India; 5 Department of Pharmacology, Kasturba Medical College, Manipal Academy of Higher Education, Manipal, Karnataka 576104 India; 6 Department of Obstetrics and Gynaecology, Kasturba Medical College, Manipal Academy of Higher Education, Manipal, Karnataka 576104 India

**Keywords:** Hypervirulent *Klebsiella pneumoniae*, Rifampicin, Antivirulence therapy, Hypermucoviscous phenotype, Scoping review

## Abstract

**Background:**

Hypervirulent/Hypermucoviscous *Klebsiella pneumoniae* (HvKp/HmKp) is associated with invasive community-acquired infections and metastatic complications, with reports of clinical failure despite in vitro susceptibility to standard antibiotics. Rifampicin has been described in experimental settings to modulate virulence by suppressing the hypermucoviscous phenotype through RNA polymerase-dependent regulation of capsule-associated genes. This scoping review aimed to systematically map the available evidence evaluating rifampicin as a potential antivirulence adjunct in HvKp/HmKp infections.

**Methods:**

This scoping review was conducted in accordance with the Joanna Briggs Institute methodology and reported following the PRISMA-ScR guidelines. A structured search of PubMed/MEDLINE, Embase, Scopus, and the Cochrane Library was performed from database inception to May 2026 using predefined terms related to hypervirulence, *Klebsiella pneumoniae*, and rifampicin/rifampin. The search was supplemented by grey literature and reference list screening. Studies evaluating rifampicin in phenotypically, genotypically, or syndromically defined HvKp were eligible, including experimental and clinical reports describing rifampicin as adjunctive or salvage therapy. Data were charted and synthesized narratively to map study characteristics, proposed mechanisms, and reported outcomes.

**Results:**

Of 1,361 records identified, 11 studies met the inclusion criteria, comprising four experimental/mechanistic studies, five full-text clinical case reports, and two conference abstracts. Experimental studies suggested that rifampicin may reduce hypermucoviscosity by suppressing capsule-associated pathways, particularly through the RpoB–*rmpA* axis, leading to reduced capsule thickness and downregulation of virulence-related genes. Additional preclinical studies showed enhanced activity when rifampicin was used in combination with zidovudine or the outer membrane-disrupting peptide SLAP-S25, with improved bacterial killing and survival in murine infection models. Clinical evidence was limited to case reports and conference abstracts in which rifampicin was used only as part of combination therapy for severe, disseminated, persistent, or difficult-to-treat HvKp/HmKp infections. Reported clinical outcomes were variable, and the independent contribution of rifampicin could not be determined.

**Conclusion:**

Current evidence suggests that rifampicin has biologically plausible antivirulence activity against HvKp/HmKp, mainly through suppression of capsule-associated hypermucoviscosity, and may have potential as an adjunct in selected severe or persistent infections. However, the available clinical evidence is sparse, uncontrolled, and heterogeneous. Rifampicin should therefore be considered hypothesis-generating rather than established therapy. Further mechanistic, pharmacokinetic, and prospective clinical studies are required to define its role, optimal combinations, dosing, safety, and clinical effectiveness in HvKp/HmKp infections.

**Supplementary Information:**

The online version contains supplementary material available at 10.1186/s12879-026-13723-7.

## Graphical Abstract

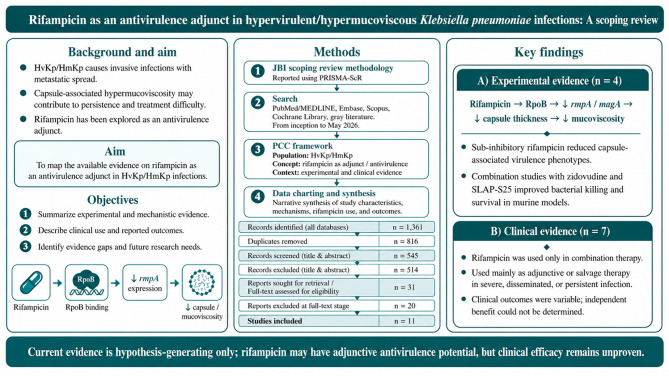

The scoping review was registered with the Open Science Framework (OSF) (Registration: https://osf.io/z5bmg).

## Introduction

Hypervirulent *Klebsiella pneumoniae* (HvKp) has emerged as a clinically important pathotype, distinct from classical *K. pneumoniae* (cKp) in its epidemiology, virulence profile, and host predilection [1]. Unlike cKp, which predominantly affects hospitalized or immunocompromised individuals, HvKp is increasingly recognized as a cause of invasive, community-acquired infections in previously healthy hosts [1, 2]. These infections frequently demonstrate metastatic dissemination, most commonly presenting as cryptogenic liver abscess, endophthalmitis, meningitis, or other deep-seated foci, and are associated with substantial morbidity and mortality [2, 3].

A commonly reported, but not universal, feature of HvKp is the hypermucoviscous phenotype, which is driven by overproduction of capsular polysaccharide and may contribute to resistance against host immune clearance [1, 2, 4]. This phenotype is commonly associated with plasmid-mediated virulence determinants, including *rmpA* and *rmpA2*, along with enhanced iron acquisition systems such as aerobactin and salmochelin [1, 4]. These factors collectively facilitate bacterial proliferation, tissue invasion, and metastatic spread. However, the identification of HvKp remains heterogeneous across studies, with variable reliance on phenotypic assays (e.g., string test) and/or genotypic markers, contributing to inconsistencies in classification and reporting [1, 4].

Historically, HvKp isolates were largely susceptible to standard antimicrobial agents. However, the increasing emergence of carbapenem-resistant hypervirulent strains (CR-HvKp) has further complicated the therapeutic landscape [5–9]. These strains arise through convergence of resistance and virulence determinants, either by acquisition of resistance genes in hypervirulent lineages or by uptake of virulence plasmids in resistant cKp clones [5–9]. Infections due to CR-HvKp are associated with limited treatment options and poor clinical outcomes [10, 11].

Importantly, clinical failure in HvKp infections is not exclusively attributable to antimicrobial resistance. Reports describe persistence of infection despite in vitro susceptibility, particularly in deep-seated infections such as liver abscesses. Proposed mechanisms include the presence of a dense capsular barrier, high bacterial burden, impaired antibiotic penetration into abscess cavities, and inoculum effects that may reduce antimicrobial efficacy [12–15]. These factors highlight the limitations of conventional bactericidal strategies in addressing HvKp-associated disease.

In this context, there is increasing interest in therapeutic approaches targeting bacterial virulence rather than bacterial viability. Antivirulence strategies aim to attenuate pathogenic mechanisms, such as capsule production or iron acquisition- without exerting direct selective pressure on bacterial survival [16–19]. Among potential candidates, rifampicin has been described in experimental studies to modulate HvKp virulence through inhibition of the β-subunit of RNA polymerase (*RpoB*), with downstream effects on capsule-associated gene expression [16, 20–23]. At sub-inhibitory concentrations, rifampicin exposure has been reported to reduce mucoviscosity and suppress transcription of virulence-associated genes, including *rmpA* [16, 20].

However, the clinical relevance of these findings remains uncertain. The proposed antivirulence effects appear dependent on intact rifampicin-*RpoB* interaction and may be attenuated by *RpoB* mutations [20]. In addition, clinical use of rifampicin in this context has been limited to isolated reports, often as adjunctive or salvage therapy in patients with persistent infection despite standard treatment [24–28]. Broader considerations, including pharmacokinetic variability, drug-drug interactions, and antimicrobial stewardship concerns, particularly in tuberculosis-endemic settings, further complicate its potential application.

To date, no scoping or systematic review has comprehensively synthesized the available literature spanning mechanistic studies and clinical reports evaluating rifampicin in HvKp infections. Given the evolving epidemiology of HvKp, the heterogeneity in its definition, and the absence of standardized antivirulence strategies, a structured mapping of existing evidence is required.

## Aim

This scoping review aims to systematically map and characterize the existing experimental and clinical evidence on the use of rifampicin as an adjunctive or salvage therapy in infections caused by HvKp/HmKp, with a focus on proposed antivirulence mechanisms, clinical applications, and reported outcomes.

## Review questions

This review was guided by the following questions:

1. What types and designs of studies have evaluated rifampicin in HvKp/HmKp?
2. What antivirulence mechanisms and phenotypic effects have been described in experimental models?
3. In which clinical scenarios has rifampicin been used, and how has it been administered?
4. What microbiological and clinical outcomes have been reported?
5. How has HvKp been defined across included studies (phenotypic versus genotypic criteria)?

## Methods

### Protocol registration

This scoping review was conducted in accordance with the Joanna Briggs Institute (JBI) methodology for scoping reviews and is reported following the Preferred Reporting Items for Systematic Reviews and Meta-Analyses extension for Scoping Reviews (PRISMA-ScR) guidelines [29].

The review protocol was prospectively registered with the Open Science Framework (OSF) [30] (Registration: https://osf.io/z5bmg).

### Eligibility criteria

Eligibility criteria were defined a priori using the Population-Concept-Context (PCC) framework.

#### Population (participants/organisms)

Studies involving *Klebsiella pneumoniae* isolates explicitly described as hypervirulent (*HvKp*) or hypermucoviscous (*HmKp*) were eligible. Identification was based on phenotypic criteria (e.g., positive string test > 5 mm) or genotypic markers associated with hypervirulence (e.g., *rmpA*, *rmpA2*, *magA*, *iucA*, *iroN*, *peg-344*). Studies focusing solely on cKp without reference to hypervirulence were excluded unless included as comparators in mechanistic studies. Because hypermucoviscosity and hypervirulence are related but not synonymous, included studies were interpreted according to the definition used by the original authors, including phenotypic-only, genotypic-confirmed, or mixed phenotypic-genotypic definitions where reported.

#### Concept

The concept of interest was rifampicin as a therapeutic or experimental intervention. This included:

- Experimental studies evaluating the effect of rifampicin on virulence-associated phenotypes (e.g., capsule production, mucoviscosity, transcriptional regulation).
- Clinical reports describing rifampicin as adjunctive or salvage therapy in HvKp infections.

#### Context

No restrictions were applied regarding study setting or geography. Eligible contexts included:

- Laboratory-based studies (in vitro assays, molecular analyses).
- Animal models (e.g., murine infection models).
- Clinical settings involving human participants.

### Information sources and search strategy

A comprehensive, multi-step search strategy was developed to identify both published and unpublished evidence on rifampicin/rifampin use in hypervirulent or hypermucoviscous *Klebsiella pneumoniae*. An initial limited search of PubMed/MEDLINE was conducted to identify relevant keywords, Medical Subject Headings/controlled vocabulary terms, and terminology used in published studies. These terms informed the final search strategy, which was adapted for each database.

A structured electronic search was performed in PubMed/MEDLINE, Embase, Scopus, and the Cochrane Library from database inception to January 2026. The initial search combined three major concepts: *Klebsiella pneumoniae*, hypervirulent/hypermucoviscous phenotype, and rifampicin/rifampin. The core search strategy included terms such as “hypervirulent,” “hypermucoviscous,” “Klebsiella pneumoniae,” “rifampicin,” and “rifampin.”

To ensure that recently published evidence was captured and to improve the sensitivity of the search, an updated search was performed in May 2026. Based on reviewer feedback and terminology identified during the initial screening, the updated strategy was broadened to include additional organism-, phenotype-, virulence-, and syndrome-related terms. The complete updated search string combined: **“hypervirulent**,**” “hypermucoviscous**,**” “mucoid**,**” “mucoviscosity**,**” “rmpA**,**” “magA**,**” “iucA**,**” and “iroB”** with organism- and syndrome-related terms including **“Klebsiella pneumoniae**,**” “K pneumoniae**,**” “Klebsiella liver abscess**,**” and “Klebsiella liver abscess syndrome**,**”** along with rifamycin-related terms **“rifampicin” and “rifampin.”** The complete database-specific search strategies, including Boolean operators, field tags, and database-specific syntax, are provided in Supplementary File 4. To enhance comprehensiveness, the electronic database search was supplemented by screening the reference lists of included studies and relevant reviews. Grey literature sources, including Google Scholar, ProQuest Dissertations and Theses, and relevant conference proceedings, were also searched. No language restrictions were applied.

### Study selection

All identified records were imported into Microsoft Excel, and duplicate entries were removed. Study selection was conducted in two stages by two independent reviewers (DN and SC). Before formal screening, both reviewers piloted the eligibility criteria on a small sample of records to ensure a common understanding of the inclusion and exclusion criteria.

First, titles and abstracts were screened independently against the predefined eligibility criteria, and clearly irrelevant records were excluded. Second, the full texts of potentially eligible studies were retrieved and independently assessed by the same two reviewers. Reasons for exclusion at the full-text stage were documented. Any disagreement during title/abstract screening or full-text assessment was resolved through discussion. If consensus could not be reached, a third reviewer (AKR) adjudicated the decision. A list of studies excluded after full-text review is provided in the supplementary file 3.

### Data charting and synthesis

Data were charted using a reviewer-developed data charting form designed specifically for this scoping review. The form was first piloted on a sample of included studies by the review team to ensure that all relevant variables were captured consistently. Based on this calibration exercise, the form was refined before final data extraction.

Data extraction was performed independently by two reviewers (DN and SC). Extracted variables for experimental studies included study design, strain characteristics, interventions, proposed mechanisms of rifampicin activity, experimental assays, and key outcomes. For clinical reports, extracted variables included patient demographics, risk factors, infection characteristics, microbiological findings including antimicrobial susceptibility testing, rifampicin dose, route, timing and duration, concomitant therapies, and reported outcomes.

After independent extraction, the completed data charting forms were compared. Discrepancies were resolved through discussion and cross-checking against the original source articles. Persistent disagreements, if any, were resolved by consultation with a third reviewer (AKR). When information was unclear or not reported in the source article, it was recorded as “not reported” rather than inferred.

Data were synthesized descriptively using a narrative approach, consistent with scoping review methodology. Findings were organized into two broad domains: (i) experimental/mechanistic evidence and (ii) clinical evidence. The objective of synthesis was to map the range and nature of available evidence rather than to evaluate comparative effectiveness. Extracted data are summarized in tabular format.

## Results

### Selection of sources of evidence

**Fig. 1:**
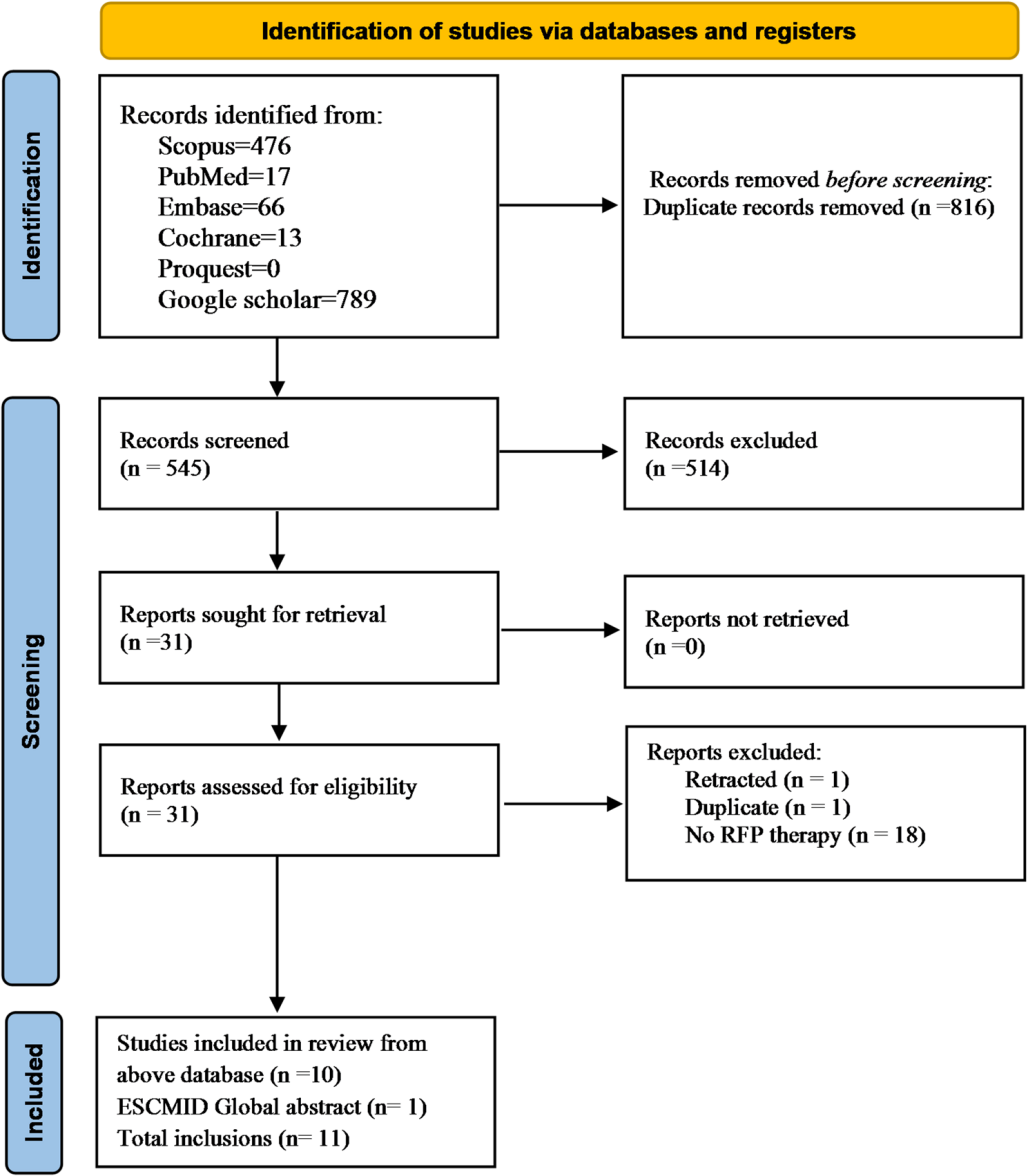
PRISMA flow diagram. Abbreviations: RFP: Rifampicin, ESCMID: European Society of Clinical Microbiology and Infectious Diseases

The literature search identified 1,361 records across all databases. After removal of 816 duplicates, 545 records underwent title and abstract screening. Of these, 514 records were excluded as irrelevant to the review question. A total of 31 reports were sought for retrieval and assessed for full-text eligibility. Following full-text assessment, 20 reports were excluded with reasons, and 11 studies met the inclusion criteria. These included four experimental/mechanistic studies, five full-text clinical case reports, and two conference abstracts (Fig. 1).

### Characteristics of included studies

Eleven studies were included in this scoping review. The included studies were published between 2019 and 2025, and all were reported in English. Experimental studies were conducted in Japan (*n* = 2) and China (*n* = 2) [16, 20, 31, 32]. Clinical case reports originated from the United States (*n* = 1), Japan (*n* = 2), Iran (*n* = 1), and China (*n* = 1) [24–27, 33]. In addition, two conference abstracts were included: one presented at CHEST 2022 [34] and one from India presented at the European Society of Clinical Microbiology and Infectious Diseases (ESCMID) Global 2024 meeting [28]. Definitions of HvKp/HmKp varied across the included studies. Experimental studies generally used characterized strains and/or virulence-associated markers, whereas clinical reports variably relied on hypermucoviscous phenotype, string-test positivity, clinical invasive syndrome, or genotypic virulence markers. Therefore, findings were interpreted according to the definition used in each study rather than assuming that all hypermucoviscous isolates were equivalent to genotypically confirmed HvKp.

### Experimental and mechanistic evidence

Four experimental studies evaluated rifampicin-based activity against HvKp or CR-HvKp using in vitro assays, molecular analyses, resistance-mutant experiments, and murine infection models (Table 1; Supplementary file 5_Table S1) [16, 20, 31, 32]. The experimental evidence broadly addressed two related themes: first, the direct antivirulence effect of rifampicin on capsule-associated phenotypes, and second, the role of rifampicin in combination-based strategies designed to improve activity against difficult-to-treat HvKp or CR-HvKp strains.

Namikawa et al. (2019) [16] evaluated the effect of rifampicin at sub-inhibitory concentrations in HvKp strains belonging to clonal group 23 (ST23, serotype K1). Rifampicin exposure was associated with reduced mucoviscosity and decreased capsular polysaccharide thickness. This was accompanied by reduced expression of capsule-associated genes, including *rmpA*, *magA*, *galF*, *wzi*, and *manC*. These effects were reported to be reversible following withdrawal of the drug.

Tohda et al. (2022) [20] examined rifampicin-resistant mutants and reported that mutations in the *RpoB* gene were associated with loss of both antimicrobial susceptibility and the reduction in mucoviscosity observed in wild-type strains. Reversion to wild-type *RpoB* restored rifampicin-associated phenotypic changes. Capsule thickness and mucoviscosity were reported to be positively correlated.

Ni et al. (2022) [31] evaluated rifampicin in combination with zidovudine against carbapenem-resistant HvKp. In vitro time–kill assays demonstrated reduced bacterial counts with combination therapy compared to monotherapy. In a murine sepsis model, survival was reported to be higher in animals receiving combination therapy compared to those receiving single agents. Resistance emergence was reported with monotherapy and appeared reduced in the combination group.

Yang et al. [32] evaluated a different combination approach using SLAP-S25, an outer membrane-disrupting peptide, with hydrophobic antibiotics including rifampicin. In this study, SLAP-S25 disrupted the Gram-negative outer membrane by targeting lipopolysaccharide, thereby increasing permeability and facilitating rifampicin entry. The combination of SLAP-S25 and rifampicin showed synergistic activity against carbapenem-resistant hypervirulent *K. pneumoniae* CRHvKP4. In a murine peritonitis/sepsis model, combination therapy improved survival and reduced bacterial burden in multiple organs compared with monotherapy or control treatment. Mechanistic experiments using permeability assays, electron microscopy, lipopolysaccharide analysis, and resistant mutants supported the role of outer membrane disruption in enhancing rifampicin activity.

**Table 1:** Summary of experimental and mechanistic evidence evaluating rifampicin-based activity against hypervirulent/hypermucoviscous *Klebsiella pneumoniae*

Study	Design/model	Strain focus	Intervention assessed	Core mechanism	Key findings
Namikawa et al. 2019 [16]	Experimental in vitro study	Four HvKp strains and one non-HvKp control	Rifampicin after screening 18 antimicrobial agents	Suppression of rmpA transcription, leading to reduced capsule-associated gene expression and mucoviscosity	Rifampicin reduced mucoviscosity and capsule thickness at subinhibitory concentrations, supporting a potential antivirulence role.
Tohda et al. 2022 [20]	Experimental in vitro mechanistic study	Wild-type OCU_HvKp1, rifampicin-resistant RpoB mutants, engineered mutant and revertants	Rifampicin exposure in wild-type, mutant and revertant strains	Anti-mucoviscous activity depends on rifampicin binding to RpoB	RpoB mutations abolished the anti-mucoviscous response; revertants regained rifampicin susceptibility, confirming target-dependent activity.
Ni et al. 2022 [31]	Experimental in vitro and murine infection study	CR-HvKp clinical isolates; CR-HvKp1 used for mechanistic and animal experiments	Zidovudine plus rifampicin combination therapy	Dual inhibition of bacterial RNA polymerase subunits with suppression of rmpA2-associated virulence	Combination therapy showed synergistic antibacterial activity, reduced virulence markers and improved survival in mice.
Yang et al. 2022 [32]	Experimental in vitro and murine peritonitis/sepsis study	*K. pneumoniae* CR-HvKp4 and other MDR Gram-negative pathogens	SLAP-S25 combined with hydrophobic antibiotics, including rifampicin	Outer membrane/LPS disruption by SLAP-S25 increases rifampicin entry	SLAP-S25 potentiated rifampicin against CRHvKP4 and improved mouse survival with reduced organ bacterial burden.

Abbreviations: ATCC, American Type Culture Collection; CR-HvKp, carbapenem-resistant Hypervirulent *Klebsiella pneumoniae*; HvKp, Hypervirulent Klebsiella pneumoniae; LPS, lipopolysaccharide; MDR, multidrug-resistant; RpoB, RNA polymerase beta subunit; ST, sequence type

### Clinical evidence

Seven studies provided clinical evidence on rifampicin use in HvKp/HmKp infections, including five full-text case reports and two conference abstracts (Table-2; Supplementary file 5_Table S2) [24–28, 33, 34]. Rifampicin was not used as monotherapy in any report; it was administered as part of combination therapy, usually in severe, disseminated, persistent, or clinically refractory infections.

Where patient-level data were reported, patients ranged from 29 to 82 years, with a predominance of male patients. Reported host risk factors included diabetes mellitus, uncontrolled hyperglycemia, immunocompromised states, splenectomy, chronic kidney disease, alcoholic liver disease, critical illness, and mechanical ventilation. The reported clinical syndromes were heterogeneous and included liver abscess with metastatic spread, emphysematous pyelonephritis with spondylitis, iliopsoas abscess, spondylodiscitis, septic pulmonary emboli, pneumonia, ventilator-associated pneumonia, bacteremia, and disseminated infection with multiple abscesses.

Rifampicin was generally introduced as an adjunctive or salvage agent after ongoing fever, persistent infection, disseminated disease, or concern for a hypermucoviscous phenotype. In reports where dosing was available, rifampicin was administered orally, with doses ranging from 450 mg once daily to 600 mg twice daily. The timing of initiation varied substantially, ranging from early use during the acute phase to delayed initiation around hospital day 20. Rifampicin duration was inconsistently reported, but where available ranged from approximately 15 days to around 50 days; total antimicrobial therapy was often longer, reaching up to 148 days in one report.

Concomitant treatment varied according to susceptibility patterns and clinical context. Rifampicin was combined with agents such as meropenem, levofloxacin, cefoperazone/sulbactam, trimethoprim-sulfamethoxazole, metronidazole, tobramycin, colistin, and other broad-spectrum antimicrobials. Source control was also frequently important, including drainage of liver or other abscesses, bronchial washings, or other supportive and procedural interventions. In the fatal invasive liver abscess syndrome case, rifampicin was added with tobramycin because of suspected hypervirulent/hypermucoviscous phenotype, but the patient deteriorated despite multiple bronchial washings, liver abscess drainage, and 27 days of antibiotic therapy.

Clinical outcomes were variable. Several reports described clinical improvement or survival after rifampicin-containing combination therapy, particularly when combined with prolonged antimicrobial therapy and source control. For example, Hamada et al. reported survival after long-term combination antibiotic therapy in a patient with iliopsoas abscess, spondylodiscitis, septic pulmonary embolism, and symmetrical peripheral gangrene due to HmKp. However, mortality was also reported, particularly in a severely immunocompromised patient with invasive liver abscess syndrome, uncontrolled diabetes, prior splenectomy, multifocal pneumonia, lung abscesses, shock, and renal failure.

Overall, the available clinical evidence suggests that rifampicin has been used mainly as an adjunctive or salvage component of combination therapy in complex HvKp/HmKp infections. However, the evidence remains limited to case reports and conference abstracts, with inconsistent reporting of rifampicin timing, duration, microbiological clearance, adverse events, and pharmacokinetic considerations.

**Table 2:** Summary of clinical evidence on rifampicin use in hypervirulent/hypermucoviscous *Klebsiella pneumoniae* infections

Study (year, country)	Patient / key risk factor(s)	Clinical syndrome	Microbiological evidence	Rifampicin-based treatment	Clinical outcome
Kawaguchi et al. 2023 [24], Japan	70-year-old female; type 2 diabetes mellitus	Emphysematous pyelonephritis complicated by pyogenic spondylitis	Blood and urine cultures positive for string-test-positive *K. pneumoniae*; magA and rmpA negative	Rifampicin 450 mg/day as adjunct to broad-spectrum therapy including meropenem and levofloxacin	Survived; inflammatory markers improved; discharged for rehabilitation on day 118
Lin et al. 2021 [25], China	29-year-old male; no major comorbidity reported	Septic arthritis with bacteremia and pneumonia	Blood culture positive for string-test-positive Hypervirulent *K. pneumoniae*; pan-susceptible isolate	Rifampicin 0.6 g every 12 h combined with meropenem 2 g every 8 h	Improved clinically within 3 days; became afebrile and was discharged
Hamada et al. 2024 [26], Japan	82-year-old male; type 2 diabetes mellitus	Liver abscess, iliopsoas abscess, spondylodiscitis, septic pulmonary embolism and symmetrical peripheral gangrene	Blood and disc aspirate grew string-test-positive Hypermucoviscous *K. pneumoniae*; isolate susceptible to evaluated antibiotics	Rifampicin 450 mg/day added early; continued with Cefoperazone/sulbactam and later meropenem-based therapy	Survived; discharged on day 113; antibiotics discontinued on day 148 after abscess resolution
Joshi et al. 2025 [27], USA	34-year-old male; splenectomy and uncontrolled diabetes mellitus	Invasive liver abscess syndrome with lung abscesses/cavitations and bacteremia	*K. pneumoniae* isolated from blood, bronchoalveolar lavage and liver abscess; high mucus phenotype suspected; resistant only to ampicillin	Rifampin added with tobramycin to a meropenem-based regimen; dose not reported	Died despite drainage, multiple antibiotics, renal replacement attempt and maximal supportive therapy
Shanbhag et al. 2024 [28], India (conference abstract)	43-year-old male; type 2 diabetes mellitus	Liver abscess with bacteremia and lung involvement	Pus and blood cultures positive; string-test-positive, pan-susceptible isolate	Rifampicin 600 mg orally twice daily with meropenem/ciprofloxacin-based therapy	Survived; Rifampicin was continued for six weeks
Sokhanvari et al. 2024 [33], Iran	39-year-old female; multiple sclerosis, psychiatric illness, bowel resection with ileostomy and mechanical ventilation	Ventilator-associated pneumonia	Tracheal culture grew carbapenem-resistant Hypervirulent ST337-K2 *K. pneumoniae*; string-test positive; iucA, rmpA2 and rmpA positive; blaSHV, blaNDM and blaOXA-48-like detected	Rifampin 10 mg/kg orally once daily for 10 days with intravenous and nebulized colistin	Tracheal culture became negative; patient discharged on day 46
Mitchell et al. 2022 [34], USA (conference abstract)	43-year-old male; type 2 diabetes mellitus with diabetic ketoacidosis	Large hepatic abscess, septic pulmonary emboli, subcutaneous abscess, prostate abscess, epididymo-orchitis, pleural effusion and bacteremia	Blood, surgical and wound cultures persistently positive for *K. pneumoniae*; Syndromic based definition of hypervirulence; detailed AST not reported	Meropenem plus rifampin after persistent positive cultures; dose and duration not reported	Clinical improvement reported

Abbreviations: AST, antimicrobial susceptibility testing; CR-HvKp, carbapenem-resistant Hypervirulent *Klebsiella pneumoniae*; DKA, diabetic ketoacidosis; HbA1c, glycated haemoglobin; HmKp, Hypermucoviscous *Klebsiella pneumoniae*; HvKp, Hypervirulent *Klebsiella pneumoniae*; ILAS, invasive liver abscess syndrome; *K. pneumoniae*, *Klebsiella pneumoniae*; NR, not reported; ST, sequence type; T2DM, type 2 diabetes mellitus; VAP, ventilator-associated pneumonia

## Discussion

This scoping review maps the currently available experimental and clinical literature evaluating rifampicin in hypervirulent or hypermucoviscous *Klebsiella pneumoniae* infections. The evidence remains limited and heterogeneous, comprising experimental in vitro studies, murine infection models, clinical case reports, and conference abstracts. Overall, the findings suggest a biologically plausible antivirulence role for rifampicin, particularly through capsule and mucoviscosity attenuation, but the available clinical evidence is insufficient to establish treatment effectiveness.

### Mechanistic evidence: the *RpoB*–*rmpA* axis and capsule-associated phenotypes

**Fig. 2:**
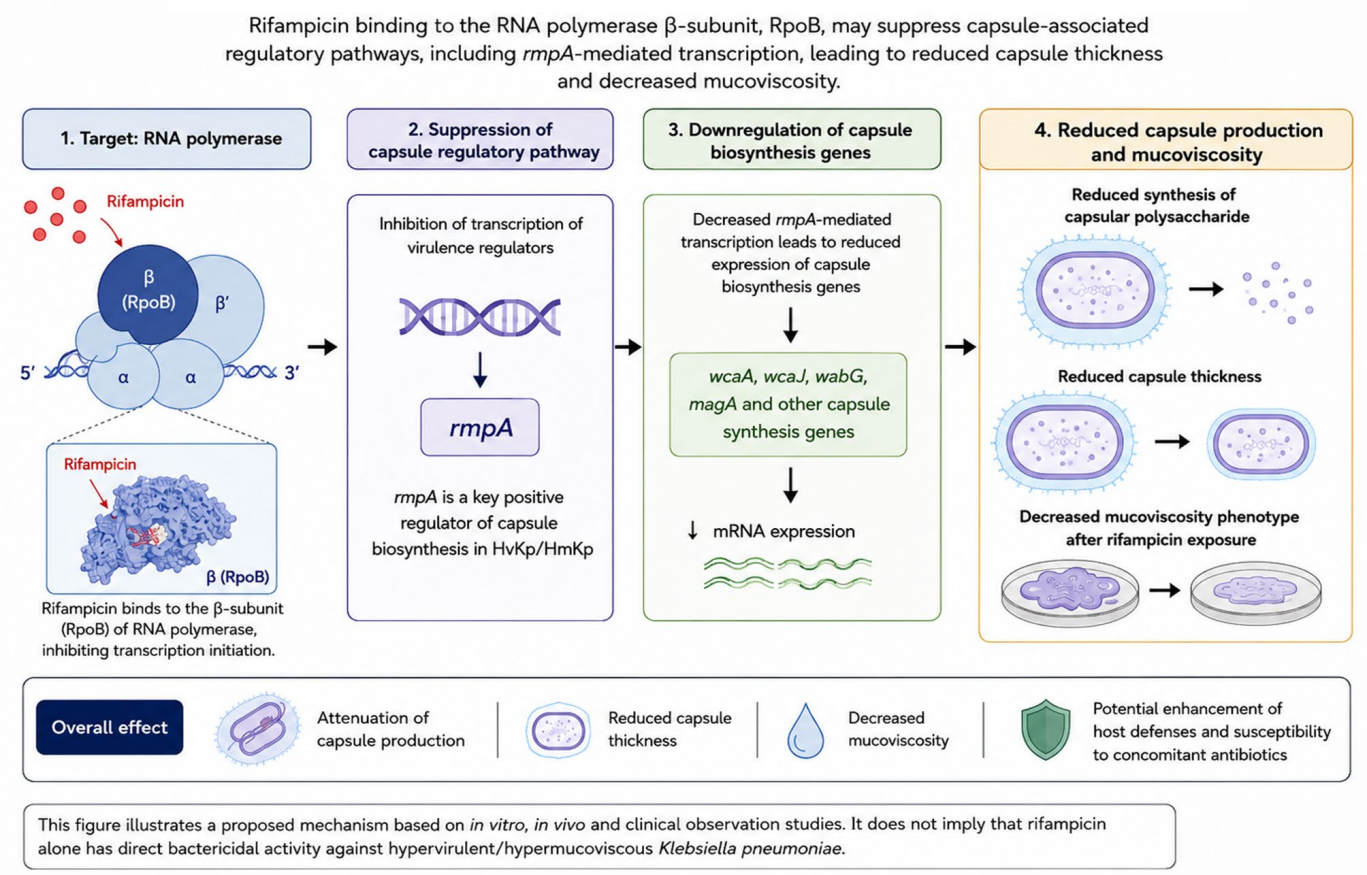
Proposed rifampicin-mediated attenuation of capsule-associated hypermucoviscosity in HvKp/HmKp. Rifampicin binding to the RNA polymerase β-subunit, RpoB, may suppress capsule-associated regulatory pathways, including rmpA-mediated transcription, leading to reduced capsule thickness and decreased mucoviscosity. This figure illustrates capsule/mucoviscosity attenuation and does not imply that rifampicin alone produces direct bactericidal activity in clinical HvKp/HmKp infection

Across the experimental studies, rifampicin exposure was consistently associated with modulation of capsule-related phenotypes. Namikawa et al. [16] demonstrated that rifampicin, even at sub-inhibitory concentrations, reduced mucoviscosity and capsular polysaccharide thickness in HvKp strains. This effect was accompanied by downregulation of capsule-associated genes, including *rmpA*, *magA*, *galF*, *wzi*, and *manC*, suggesting that rifampicin may attenuate virulence-associated traits by suppressing capsule production pathways. Figure 2 outlines the proposed mechanism of rifampicin-mediated virulence attenuation through *rmpA* downregulation and capsule inhibition in HvKp.

Tohda et al. [20] further strengthened this mechanistic framework by showing that rifampicin’s anti-mucoviscous effect depends on its interaction with the RNA polymerase beta subunit, RpoB. Rifampicin-resistant mutants carrying *rpoB* mutations lost the anti-mucoviscous response, whereas revertants with restored wild-type *rpoB* regained susceptibility to rifampicin-mediated mucoviscosity reduction. These findings suggest that rifampicin’s antivirulence effect is target-dependent and linked to the RpoB–*rmpA*–capsule pathway. However, the same findings also highlight a potential limitation: this effect may be unstable under selective pressure, particularly when *rpoB*-mediated resistance emerges.

### Rifampicin in experimental combination strategies

Experimental studies extend the concept of rifampicin from a direct antivirulence agent to a component of rational combination therapy. Ni et al. [31] evaluated rifampicin in combination with zidovudine against CR-HvKp. This combination was proposed to act through dual targeting of bacterial RNA polymerase subunits, with rifampicin acting on RpoB and zidovudine on RpoC. The combination reduced bacterial burden, suppressed *rmpA2*-associated mucoviscosity, delayed resistance development, and improved survival in a murine sepsis model compared with monotherapy. These findings suggest that rifampicin may have greater utility when paired with agents that act on complementary bacterial targets rather than when used alone.

Yang et al. [32] provided another combination-based approach by pairing rifampicin with SLAP-S25, an outer membrane-disrupting peptide. In this study, SLAP-S25 disrupted the Gram-negative outer membrane by targeting lipopolysaccharide, thereby increasing permeability and facilitating the entry of hydrophobic antibiotics such as rifampicin. The combination showed synergistic activity against CR-HvKp and improved survival with reduced organ bacterial burden in a murine peritonitis/sepsis model. This supports the concept that rifampicin activity against Gram-negative pathogens may be enhanced when permeability barriers are overcome.

A related experimental report by Ni et al. [35] was not considered as a separate included study because of overlap with the data reported in Ni et al. [31]. Nevertheless, it provides additional mechanistic context for the rifampicin–zidovudine combination. The report suggests that this combination may exert effects beyond direct antibacterial activity, including attenuation of inflammatory responses and partial restoration of gut microbiota alterations in experimental CR-HvKp infection. These findings indicate that rifampicin-based combination therapy may potentially influence host–pathogen and gut–immune interactions in addition to bacterial killing. However, these observations remain preclinical and should be interpreted cautiously until validated in independent experimental and clinical studies.

### Case-based synthesis: timing, dosing, duration, and the primacy of source control

The included cases describe the use of rifampicin in two broad clinical contexts: (i) early adjunctive use when a hypermucoviscous phenotype is suspected, often in abscess-dominant disease, and (ii) later introduction as a salvage adjunct following apparent non-response to antimicrobial therapy despite in vitro susceptibility. These patterns reflect clinician-reported decision-making in individual cases rather than prospectively evaluated treatment strategies.

Joshi et al. [27] described escalation to include rifampicin and tobramycin in the setting of suspected HvKp/high-mucoviscosity phenotype; however, the patient died despite combination therapy. The patient was critically ill and required multiple vasopressors. As previously noted, shock states may be associated with splanchnic hypoperfusion and gastrointestinal dysmotility, which can result in variable oral drug absorption [36], potentially leading to reduced rifampicin exposure. This case illustrates that, in critically ill patients, multiple factors including pharmacokinetic variability, delayed or incomplete source control, and high pathogen burden may influence outcomes. Accordingly, rifampicin use in this context should be interpreted as part of a broader management strategy rather than as a substitute for timely source control and appropriate antimicrobial therapy.

Hamada et al. [26] describes a contrasting clinical course. Rifampicin was introduced during the acute phase, reportedly based on a presumed anti-mucoviscosity rationale, and subsequent antimicrobial regimens were modified as individual infectious foci evolved differently. While the liver abscess improved, the iliopsoas infection persisted and required further antibiotic adjustments before resolution. Notably, rifampicin was not continued throughout the full treatment course, which may suggest that its use was temporally limited in this case; however, the specific rationale for discontinuation was not explicitly described.

Kawaguchi et al. [24] highlights diagnostic heterogeneity. Hypermucoviscosity was observed despite the absence of classical virulence genes. Rifampicin was used in combination with meropenem and levofloxacin and later briefly with co-trimoxazole, followed by discontinuation, with reported improvement in inflammatory markers. This case suggests that phenotypic features, such as string test positivity or clinical suspicion of a hypermucoviscous phenotype, may influence treatment decisions in the absence of genotypic confirmation. It also underscores that the absence of *magA/rmpA* does not necessarily exclude a clinically invasive phenotype, reinforcing the need for standardized definitions and reporting.

Lin et al. [25] reported persistence of fever across multiple antimicrobial regimens despite in vitro susceptibility, followed by clinical improvement after initiation of meropenem in combination with rifampicin. The report also discusses diabetes and hyperglycemia as potential contributors to invasive disease. However, given the concurrent use of combination therapy and the absence of a comparator, the independent contribution of rifampicin to the observed clinical course cannot be determined.

Sokhanvari et al. [33] reported microbiological clearance in ventilator-associated pneumonia due to CR-HvKp treated with rifampicin plus colistin. Mitchell et al. [34], although limited to abstract-level data, described clinical improvement after rifampicin was added to meropenem in disseminated infection.

Finally, the ESCMID 2024 conference abstract (Shanbhag et al.) [28], although limited by abstract-level reporting, describes prolonged use of rifampicin as part of combination therapy in a refractory invasive presentation, with survival noted. However, detailed clinical, microbiological, and pharmacologic data were not available.

### HvKp definition variability and phenotype–genotype discordance

A key observation from this review is the variability in how HvKp or HmKp was defined across studies. Experimental studies generally used well-characterized strains and molecular markers, whereas clinical reports often relied on phenotypic criteria such as string-test positivity or syndromic features of invasive disease. Genotypic confirmation was not consistently reported. Defining HvKp isolates based on the string test alone has limited specificity compared with genotypic markers, as described by Russo et al. [37].

This variability complicates interpretation. Kawaguchi et al. [24] reported hypermucoviscosity despite absence of classical *magA* and *rmpA* markers, suggesting phenotype–genotype discordance. Conversely, Sokhanvari et al. [33] described a carbapenem-resistant hypervirulent ST337-K2 isolate with virulence markers including *iucA*, *rmpA2*, and *rmpA*. These differences indicate that the biological basis of hypervirulence may vary across isolates, and rifampicin’s proposed antivirulence effect may not apply uniformly to all organisms labelled as HvKp or HmKp. Future studies should use standardized definitions incorporating clinical syndrome, phenotypic markers, genotypic virulence determinants, and resistance profiles.

### Host factors: diabetes and invasive disease patterns

Diabetes mellitus was frequently reported among the clinical cases [24, 26–28, 34]. This is consistent with the broader understanding that diabetes and hyperglycemia increase susceptibility to invasive HvKp infection, particularly liver abscess and metastatic spread. Hyperglycemia may impair neutrophil function and host immune responses, potentially contributing to dissemination and persistent infection.

Other host factors, including splenectomy, critical illness, mechanical ventilation, and immunocompromised status, were also reported [27, 33]. These factors may influence both disease severity and response to treatment. However, the available clinical evidence is insufficient to determine whether rifampicin-containing regimens have differential effects in specific host-risk groups.

### Context from broader literature on rifampicin combination therapy

Although not specific to HvKp, existing literature on rifampicin use in combination regimens for multidrug-resistant Gram-negative infections provides relevant context. The ESCMID guidelines [38] indicate that combination therapy may be considered in severe infections, although optimal regimens remain uncertain.

Experimental studies have also described synergistic activity of rifampicin when used with other antimicrobial agents, potentially related to enhanced intracellular penetration or complementary mechanisms of action [39]. While these findings are not specific to HvKp, they may offer a conceptual framework for understanding the use of rifampicin in combination regimens in the reported cases. However, extrapolation to HvKp-specific contexts should be made with caution.

### Clinical interpretation: limitations of susceptibility-based treatment paradigms

A recurring theme across the clinical reports is that in vitro susceptibility does not always translate into rapid clinical response. Persistent infection despite apparently active antimicrobials may reflect high bacterial burden, deep-seated abscesses, impaired antibiotic penetration, metastatic foci, and the protective effect of a dense capsular matrix [12–15]. These features are particularly relevant in HvKp/HmKp infections, where abscess formation and dissemination are common.

Within this context, rifampicin has been used as an adjunctive agent when clinicians suspected that conventional therapy alone was insufficient. However, the available reports do not allow separation of rifampicin’s effect from that of concomitant antibiotics, source control, and supportive care. Therefore, rifampicin should not be interpreted as a replacement for appropriate first-line antimicrobial therapy or timely drainage of infected collections. Instead, the current evidence supports only a hypothesis-generating role for rifampicin as a possible adjunct in selected severe or persistent infections.

### Pharmacologic and translational considerations

The potential use of rifampicin as an adjunct in HvKp infections raises important pharmacologic and stewardship concerns. Rifampicin has complex pharmacokinetics, and oral absorption may be unreliable in critically ill patients, particularly in shock states, gastrointestinal dysfunction, or altered perfusion [36]. This is relevant to severe HvKp infections, where patients may require vasopressors, organ support, or intensive care.

Rifampicin is also a potent inducer of hepatic drug-metabolizing enzymes, creating a high risk of drug–drug interactions. These interactions may affect co-administered antimicrobials, anticoagulants, antifungals, immunosuppressants, and other critical medications. In addition, resistance can emerge rapidly when rifampicin is used alone, and experimental evidence indicates that *rpoB* mutations may abolish both antibacterial and anti-mucoviscous effects [20]. Therefore, rifampicin should be considered only in combination regimens and with careful attention to susceptibility, drug interactions, dosing, duration, and resistance emergence.

In tuberculosis-endemic settings, inappropriate or widespread use of rifampicin also raises public health concerns. Any expanded use outside established indications should therefore be guided by antimicrobial stewardship principles and supported by stronger clinical evidence.

### Reporting gaps and implications for future research

This review identified several important reporting gaps. Clinical reports often lacked consistent information on rifampicin dose, route, timing, duration, pharmacokinetic monitoring, adverse events, resistance emergence, and microbiological clearance. Definitions of HvKp/HmKp were inconsistent, and genotypic markers were not uniformly reported. Conference abstracts provided useful signals but were limited by incomplete methodological and outcome details.

Future studies should use standardized reporting frameworks for HvKp/HmKp infections. At minimum, reports should include host risk factors, infection sites, source-control procedures, phenotypic findings, virulence markers, antimicrobial susceptibility, rifampicin dose and route, timing of initiation, duration of treatment, concomitant antibiotics, microbiological clearance, adverse events, and clinical outcome. Experimental studies should evaluate rifampicin-based combinations across diverse HvKp lineages, including carbapenem-resistant and non-carbapenem-resistant strains. Prospective clinical studies are needed to determine whether rifampicin-containing regimens provide any measurable benefit over optimized standard therapy and source control.

### Strengths and limitations

This scoping review provides a structured mapping of experimental and clinical evidence evaluating rifampicin in HvKp/HmKp infections, using methodology aligned with JBI and PRISMA-ScR guidance [29]. By integrating mechanistic studies, animal models, case reports, and conference abstracts, it offers a broad overview of the current evidence landscape.

However, several limitations must be acknowledged. The number of included studies was small, and the clinical evidence consisted only of case reports and conference abstracts. The included studies were heterogeneous in design, definitions, strain characteristics, rifampicin regimens, concomitant therapy, and outcome reporting. As expected for a scoping review, no formal risk-of-bias assessment or quantitative synthesis was performed. In addition, although the search strategy was expanded and updated, some relevant studies using alternative terminology may have been missed.

## Conclusion

Overall, the available evidence suggests that rifampicin has a biologically plausible antivirulence effect against HvKp/HmKp through suppression of capsule-associated mucoviscosity, particularly via the RpoB–*rmpA* pathway. Combination-based experimental studies suggest that rifampicin may also have enhanced activity when combined with agents such as zidovudine or outer membrane disruptors. However, the clinical evidence remains sparse, uncontrolled, and insufficient to establish efficacy. At present, rifampicin should be viewed as a potential adjunctive or salvage component in selected severe HvKp/HmKp infections, rather than as a validated treatment strategy. Further mechanistic, pharmacologic, and prospective clinical studies are needed before rifampicin-containing regimens can be recommended more broadly.

## Supplementary Information

Below is the link to the electronic supplementary material.

Supplementary Material 1

Supplementary Material 2

Supplementary Material 3

Supplementary Material 4

Supplementary Material 5

## Data Availability

All data generated or charted during this scoping review are provided within the manuscript and supplementary files. The data charting form used for extraction is provided as Supplementary File (1) The completed extracted dataset of included studies is provided as Supplementary File (2) The list of studies excluded after full-text review, along with reasons for exclusion, is provided as Supplementary File 3.

## Abbreviations

AST: Antimicrobial Susceptibility Testing
ATCC: American Type Culture Collection
BAL: Bronchoalveolar Lavage
CFU: Colony-Forming Units
CG: Clonal Group
CR-HvKp: Carbapenem-Resistant Hypervirulent Klebsiella pneumoniae
CPZ/SBT: Cefoperazone/Sulbactam
cKp: Classical Klebsiella pneumoniae
ESCMID: European Society of Clinical Microbiology and Infectious Diseases
Hb: Haemoglobin
HbA1c: Glycated Haemoglobin
HmKp: Hypermucoviscous Klebsiella pneumoniae
HvKp: Hypervirulent Klebsiella pneumoniae
ICU: Intensive Care Unit
ITC: Isothermal Titration Calorimetry
JBI: Joanna Briggs Institute
K: Lysine (also used as association constant in ITC context)
MIC: Minimum Inhibitory Concentration
NA: Not Available
OSF: Open Science Framework
PCC: Population, Concept, and Context
PK/PD: Pharmacokinetic/Pharmacodynamic
PRISMA-ScR: Preferred Reporting Items for Systematic Reviews and Meta-Analyses Extension for Scoping Reviews
q12h: Every 12 h
qPCR: Quantitative Polymerase Chain Reaction
qRT-PCR: Quantitative Reverse Transcription Polymerase Chain Reaction
RBC: Red Blood Cells
RFP: Rifampicin
RNA: Ribonucleic Acid
rmpA: Regulator of Mucoid Phenotype A
RRDR: Rifampicin Resistance-Determining Region
RpoB: RNA Polymerase Subunit Beta
RpoC: RNA Polymerase Subunit Beta′
SP: Status Post
ST: Sequence Type
T2DM: Type 2 Diabetes Mellitus
TID: Three Times Daily
WGS: Whole-Genome Sequencing
ZDV: Zidovudine
