# Supplementary material for: Rifampicin as an antivirulence adjunct in hypervirulent/hypermucoviscous Klebsiella pneumoniae infections: a scoping review
Source: BMC Infect Dis. 2026 Jun 5;26:1392. doi: 10.1186/s12879-026-13723-7 (PMC13397727; doi:10.1186/s12879-026-13723-7)
Supplement: Supplementary file 1 — Supplementary Material 1 [file 12879_2026_13723_MOESM1_ESM.pdf]

Supplementary File 1. Data charting form used for extraction

Rifampicin as an antivirulence adjunct in hypervirulent/hypermucoviscous Klebsiella pneumoniae infections: A scoping review

Purpose and use. This reviewer-developed data charting form was used to extract information from eligible experimental/mechanistic studies and clinical reports. The form was piloted before final extraction and refined to ensure consistency. Data were charted independently by two reviewers, compared after extraction, and discrepancies were resolved through discussion and, when required, adjudication by a third reviewer. Information not available in the source article was recorded as "NR" (not reported).

A. Experimental/mechanistic studies

Domain	Data item to be charted	Extraction guidance / definition	Response / value charted
Experimental study characteristics	Study aim/objective	Extract the stated study aim or the aim relevant to rifampicin and HvKp/HmKp.	
Experimental study characteristics	Study design/model	Record in vitro study, in vivo murine model, combined in vitro/in vivo study, resistance-mutant experiment, or other design.	
Strain/model details	Number of strains or isolates tested	Record total number and whether all or selected strains were used for mechanistic/in vivo studies.	
Strain/model details	Organism and strain names	Record strain names/IDs and whether hypervirulent, hypermucoviscous, carbapenem-resistant, control strain, mutant, or revertant.	
Strain/model details	Genotypic/phenotypic characteristics	Record sequence type, capsule type/serotype, resistance profile, string test, virulence genes, and relevant mutations when reported.	
Intervention	Rifampicin exposure	Record rifampicin concentration, dose, route, frequency, timing, and whether sub-inhibitory concentrations were used.	
Intervention	Combination agent(s)	Record other agents combined with rifampicin, including zidovudine, SLAP-S25, hydrophobic antibiotics, or other antimicrobials.	
Intervention	Comparator/control	Record untreated control, monotherapy arm, vehicle/PBS, wild-type strain, mutant, or other comparator.	
Methods/assays	Antibacterial susceptibility and synergy assays	Record MIC, FIC/checkerboard, time-kill, resistance development, or other antibacterial assays.	
Methods/assays	Virulence/mucoviscosity assays	Record mucoviscosity measurement, capsule thickness, string test, polysaccharide/capsule assays, or microscopy.	
Methods/assays	Molecular assays	Record qPCR/qRT-PCR, gene expression analysis, sequencing, mutagenesis, docking, ITC, or other molecular methods.	
Methods/assays	Animal model details	If applicable, record animal species/strain, infection model, inoculum, group size, route of infection, treatment timing, dose, and follow-up period.	
Mechanism	Proposed mechanism	Summarize the proposed mechanism, including RpoB binding, rmpA/magA suppression, capsule reduction, RpoC targeting, outer membrane disruption, LPS effects, or permeability changes.	
Outcomes	Main experimental findings	Record key quantitative or qualitative findings relevant to rifampicin activity, mucoviscosity, capsule, bacterial killing, resistance, or survival.	
Outcomes	Safety/toxicity findings	Record cytotoxicity, hemolysis, histology, animal toxicity, or adverse experimental findings when reported.	
Outcomes	Limitations noted for extraction	Record important limitations or applicability concerns, such as selected strains, preclinical model, or incomplete dosing data.	

B. Clinical studies and case reports

Domain	Data item to be charted	Extraction guidance / definition	Response / value charted
Clinical study characteristics	Study type and setting	Record case report, case series, conference abstract, hospital/country, and year.	
Patient characteristics	Age and sex	Record patient age and sex; if not reported, enter NR.	

Domain	Data item to be charted	Extraction guidance / definition	Response / value charted
Patient characteristics	Relevant risk factors/comorbidities	Record diabetes mellitus, immunosuppression, splenectomy, critical illness, chronic kidney disease, liver disease, mechanical ventilation, or other risk factors.	
Clinical syndrome	Infection site(s)/syndrome	Record clinical diagnosis and infection sites such as liver abscess, pneumonia, bacteremia, spondylitis, pyelonephritis, septic arthritis, metastatic infection, or ventilator-associated pneumonia.	
Clinical syndrome	Severity and complications	Record shock, organ failure, metastatic spread, persistent bacteremia, ICU admission, invasive procedures, or other complications.	
Microbiology	Positive specimen(s)	Record sample source(s), such as blood, urine, pus/abscess aspirate, sputum, bronchoalveolar lavage, or tissue.	
Microbiology	Organism and HvKp/HmKp evidence	Record string-test result, hypermucoviscous phenotype, virulence genes, sequence type, capsule type, and whether HvKp/HmKp was defined phenotypically, genotypically, or clinically.	
Microbiology	Antimicrobial susceptibility/resistance	Record susceptibility profile, carbapenem resistance, ESBL/carbapenemase status, and rifampicin susceptibility if reported.	
Rifampicin regimen	Dose	Record rifampicin dose in mg and frequency; enter NR if not reported.	
Rifampicin regimen	Route of administration	Record oral, intravenous, or NR.	
Rifampicin regimen	Timing of initiation	Record hospital day, illness day, or clinical reason for starting rifampicin, such as persistent fever, refractory infection, hypermucoviscous phenotype, or salvage therapy.	
Rifampicin regimen	Duration of rifampicin therapy	Record number of days/weeks or planned/actual duration.	
Concomitant therapy	Other antimicrobial therapy	Record all antimicrobials used before and with rifampicin, including carbapenems, fluoroquinolones, aminoglycosides, colistin, tigecycline, cephalosporins, or others.	
Concomitant therapy	Source control and supportive care	Record drainage, surgery, debridement, bronchial washings, renal replacement therapy, ICU care, ventilation, or other interventions.	
Outcome	Clinical response	Record improvement, persistent infection, recurrence, discharge, rehabilitation, or death.	
Outcome	Microbiological response	Record culture clearance, persistent positivity, or NR.	
Outcome	Adverse events/drug interactions	Record rifampicin-related adverse events or drug interactions when reported; otherwise NR.	
Outcome	Attribution caveat	Record whether rifampicin was used in combination and whether its independent contribution could be determined.	
