# Supplementary material for: Rifampicin as an antivirulence adjunct in hypervirulent/hypermucoviscous Klebsiella pneumoniae infections: a scoping review
Source: BMC Infect Dis. 2026 Jun 5;26:1392. doi: 10.1186/s12879-026-13723-7 (PMC13397727; doi:10.1186/s12879-026-13723-7)
Supplement: Supplementary file 3 — Supplementary Material 3 [file 12879_2026_13723_MOESM3_ESM.pdf]

Supplementary File 3. Studies excluded after full-text review with reasons

This supplementary file lists reports excluded after full-text assessment and the primary reason for exclusion.

Sl. no.	Study title	Reason for exclusion
1	High prevalence of blaCTX-M-15 and nosocomial transmission of hypervirulent epidemic clones of Klebsiella pneumoniae at a tertiary hospital in Ethiopia	No rifampicin/rifampin therapy reported
2	Genomic analysis of virulent, multidrug-resistant Klebsiella pneumoniae and Klebsiella oxytoca from bloodstream infections, South Africa	No rifampicin/rifampin therapy reported
3	Lowering mortality risk in CR-HvKP infection in intestinal immunohistological and microbiota restoration	Duplicate/overlapping report of an included study
4	Whole-genome sequence of carbapenem-resistant hypermucoviscous Klebsiella pneumoniae K2-ST375 with blaNDM-harbouring conjugative IncX3 and pLVPK-like virulence plasmids from a patient in China	No rifampicin/rifampin therapy reported
5	Severe disseminated infection by hypermucoviscous Klebsiella pneumoniae successfully treated by intensive therapy with continuous hemodiafiltration using AN69ST: A case report and review of the literature	Retracted article
6	Pathogenetic conditions of treatment of infections caused by antibiotic-resistant strains Klebsiella pneumoniae	No rifampicin/rifampin therapy reported
7	Duplication of the chromosomal blaSHV-11 gene in a clinical hypermutable strain of Klebsiella pneumoniae	No rifampicin/rifampin therapy reported
8	Clinical carbapenem-resistant Klebsiella pneumoniae (CRKP) strains readily develop beta-lactam/beta-lactamase resistance and are hypervirulent	No rifampicin/rifampin therapy reported
9	Whole-genome sequencing of multidrug-resistant Klebsiella pneumoniae with capsular serotype K2 isolates from mink in China	No rifampicin/rifampin therapy reported
10	High prevalence of blaCTX-M-15 and nosocomial transmission of hypervirulent epidemic clones of Klebsiella pneumoniae at a tertiary hospital in Ethiopia	No rifampicin/rifampin therapy reported
11	Duplication of the chromosomal blaSHV-11 gene in a clinical hypermutable strain of Klebsiella pneumoniae	No rifampicin/rifampin therapy reported
12	Whole-genome sequence of carbapenem-resistant hypermucoviscous Klebsiella pneumoniae K2-ST375 with blaNDM-harbouring conjugative IncX3 and pLVPK-like virulence plasmids from a patient in China	No rifampicin/rifampin therapy reported
13	Clinical and phenotypic differences between classic and hypervirulent Klebsiella pneumoniae: An emerging and under-recognized pathogenic variant	No rifampicin/rifampin therapy reported
14	Community-acquired Klebsiella pneumoniae K1 serotype invasive liver abscess with bacteremia and endophthalmitis	No rifampicin/rifampin therapy reported
15	A case of invasive infection caused by a highly virulent strain of Klebsiella pneumoniae displaying hypermucoviscosity in a patient with hepatic involvement without liver abscess	No rifampicin/rifampin therapy reported
16	Identification and Management of the Hypervirulent Invasive Klebsiella pneumoniae Syndrome: A Unique and Distinct Clinical Entity	No rifampicin/rifampin therapy reported
17	Klebsiella distinctive syndrome presenting with muscular abscess and osteomyelitis: Case report	No rifampicin/rifampin therapy reported
18	Case report: Trauma-induced Klebsiella pneumoniae invasive syndrome presenting with liver abscess, lung abscess, endophthalmitis, and purulent meningitis	No rifampicin/rifampin therapy reported
19	Klebsiella pneumoniae liver abscess: A case series of six Asian patients	No rifampicin/rifampin therapy reported
20	Emerging multiorgan Klebsiella pneumoniae invasive syndrome leading to septic shock: A case report and review of the literature	No rifampicin/rifampin therapy reported

Abbreviations: CR-HvKP, carbapenem-resistant hypervirulent Klebsiella pneumoniae; CRKP, carbapenem-resistant Klebsiella pneumoniae; RFP, rifampicin/rifampin.
