# Supplementary material for: Rifampicin as an antivirulence adjunct in hypervirulent/hypermucoviscous Klebsiella pneumoniae infections: a scoping review
Source: BMC Infect Dis. 2026 Jun 5;26:1392. doi: 10.1186/s12879-026-13723-7 (PMC13397727; doi:10.1186/s12879-026-13723-7)
Supplement: Supplementary file 4 — Supplementary Material 4 [file 12879_2026_13723_MOESM4_ESM.pdf]

Supplementary File 4. Search strategies for major databases

PubMed/Medline	(hypervirulent[tiab] OR hypermucoviscous[tiab] OR mucoid[tiab] OR mucoviscosity[tiab] OR rmpa[tiab] OR maga[tiab] OR iuca[tiab] OR irob[tiab]) AND ("klebsiella pneumoniae"[tiab] OR "k pneumoniae"[tiab] OR "klebsiella liver abscess"[tiab] OR "klebsiella liver abscess syndrome"[tiab]) AND (rifampicin[all] OR rifampin[all])
Embase	(hypervirulent:ti,ab OR hypermucoviscous:ti,ab OR mucoid:ti,ab OR mucoviscosity:ti,ab OR rmpa:ti,ab OR maga:ti,ab OR iuca:ti,ab OR irob:ti,ab) AND ('klebsiella pneumoniae':ti,ab OR 'k pneumoniae':ti,ab OR 'klebsiella liver abscess':ti,ab OR 'klebsiella liver abscess syndrome':ti,ab) AND (rifampicin OR rifampin)
Scopus	(TITLE-ABS(hypervirulent) OR TITLE-ABS(hypermucoviscous) OR TITLE-ABS(mucoid) OR TITLE-ABS(mucoviscosity) OR TITLE-ABS(rmpa) OR TITLE-ABS(maga) OR TITLE-ABS(iuca) OR TITLE-ABS(irob)) AND (TITLE-ABS("klebsiella pneumoniae") OR TITLE-ABS("k pneumoniae") OR TITLE-ABS("klebsiella liver abscess") OR TITLE-ABS("klebsiella liver abscess syndrome")) AND (ALL(rifampicin) OR ALL(rifampin))
Cochrane library	(hypervirulent:ti,ab OR hypermucoviscous:ti,ab OR mucoid:ti,ab OR mucoviscosity:ti,ab OR rmpa:ti,ab OR maga:ti,ab OR iuca:ti,ab OR irob:ti,ab) AND ("klebsiella pneumoniae":ti,ab OR "k pneumoniae":ti,ab OR "klebsiella liver abscess":ti,ab OR "klebsiella liver abscess syndrome":ti,ab) AND (rifampicin OR rifampin)
