# Supplementary material for: Rifampicin as an antivirulence adjunct in hypervirulent/hypermucoviscous Klebsiella pneumoniae infections: a scoping review
Source: BMC Infect Dis. 2026 Jun 5;26:1392. doi: 10.1186/s12879-026-13723-7 (PMC13397727; doi:10.1186/s12879-026-13723-7)
Supplement: Supplementary file 5 — Supplementary Material 5 [file 12879_2026_13723_MOESM5_ESM.pdf]

Supplementary Table S1. Detailed extraction of study characteristics, interventions, mechanisms and outcomes

Study	Aim	Strains/model details	Intervention/dosing details	Methods/assays	Detailed mechanism	Detailed outcomes
Namikawa et al. [16]	Identify an antimicrobial agent that suppresses hvKp hypermucoviscosity and evaluate rifampicin as an antivirulence candidate.	Five strains: four hvKp isolates (OCU_hvKP1-4) and one non-hvKp control (K. pneumoniae ATCC 700603). hvKp isolates were ST23/CG23-I; control lacked magA and rmpA.	Eighteen antimicrobial agents screened. Rifampicin was assessed mainly at subinhibitory concentrations; 8 ug/mL used in several capsule and expression experiments.	MIC testing, extracellular polysaccharide viscosity, India ink capsule microscopy, qPCR of rmpA, magA, galF, wzi, manC, iucA and iroN, and rifampicin addition/removal time-course experiments.	Rifampicin suppressed rmpA transcription and downregulated capsule polysaccharide synthesis genes, reducing capsule production and mucoviscosity. The effect was reversible.	Rifampicin reduced mucoviscosity in all four hvKp isolates, reduced capsule thickness to near non-hvKp levels, decreased rmpA, magA, galF, wzi and manC expression, and did not significantly suppress iucA or iroN.
Tohda et al. [20]	Determine whether rifampicin anti-mucoviscous activity is mediated through binding to RpoB.	Twelve experimental strains: wild-type OCU_hvKP1, five spontaneous rifampicin-resistant mutants (R1-R5), one engineered RpoB mutant (R6) and five wild-type rpoB revertants (R1'-R5').	Rifampicin exposure at 8 ug/mL in comparative experiments; rifampicin-resistant mutants selected on rifampicin-containing agar. Allelic exchange used to confirm rpoB involvement.	Whole-genome sequencing, MIC testing, growth curves, Ostwald viscometer mucoviscosity assay, two-step rpoB allelic exchange, qRT-PCR for rmpA/magA, and India ink capsule microscopy.	Rifampicin binding to RpoB was required for anti-mucoviscous activity. rpoB mutations conferred resistance to both growth-inhibitory and anti-mucoviscous effects; wild-type rpoB restoration restored response.	R1-R5 had non-synonymous rpoB mutations and rifampicin MICs >512 ug/mL versus 32 ug/mL in wild type. Mutants had little/no mucoviscosity reduction with rifampicin; revertants regained reduction in mucoviscosity, rmpA/magA expression and capsule thickness.
Ni et al. [31]	Evaluate zidovudine plus rifampicin for CR-HvKp and assess synergy, resistance development, toxicity and mechanism.	Thirty CR-HvKp clinical isolates were used for MIC testing; one CR-HvKP1 strain was used for time-kill, resistance, mechanistic and murine sepsis experiments.	Zidovudine and rifampicin tested alone and in combination; reported synergistic ratio 8:5. In the murine sepsis model, mice infected with 3 x 10^7 CFU CR-HvKP1 received oral drug treatment every 12 h.	MIC testing, time-kill assay, murine sepsis survival model, resistance serial passage, haemolysis and HepG2 toxicity assays, liver/kidney histology, ITC, molecular docking and qRT-PCR of rmpA2.	Zidovudine was proposed to bind RpoC and rifampicin RpoB. Dual RNA polymerase targeting inhibited transcription and reduced rmpA2 expression and mucoviscosity.	Zidovudine MIC 1.25-5 ug/mL; rifampicin MIC 16-64 ug/mL; combination MIC 0.25/0.15625 to 4/2.5 ug/mL. Combination eradicated CR-HvKP1 in 24 h and achieved 100% mouse survival compared with 0% control/ZDV and 34% rifampicin. Combination limited resistance and showed low toxicity.
Yang et al. [36]	Investigate synergy between SLAP-S25 and hydrophobic antibiotics, including rifampicin, against MDR Gram-negative pathogens and CR-HvKp.	Five representative Gram-negative strains, including K. pneumoniae CRHvKP4, A. baumannii AB176, P. aeruginosa PAO1/PA16-2, E. cloacae EC20 and colistin-resistant E. coli B2. K. pneumoniae ATCC 43816 and LPS/OM mutants were used for mechanism.	Chequerboard assays tested SLAP-S25 plus hydrophobic antibiotics. In mice, BALB/c groups (n=6) received IP PBS, SLAP-S25, rifampicin or SLAP-S25 plus rifampicin 1 h after infection with about 7.5 x 10^6 CFU CRHvKP4.	Chequerboard MIC/FIC assays, growth curves, time-kill assays, FE-SEM, confocal membrane-integrity staining, NPN OM permeability assay, LPS extraction/SDS-PAGE, resistance development and organ bacterial burden analysis.	SLAP-S25 disrupted the Gram-negative OM by targeting/removing LPS, increasing permeability and facilitating intracellular entry of hydrophobic antibiotics such as rifampicin.	With 4 mg/L SLAP-S25, rifampicin activity against CRHvKP4 increased at least 64-fold. SLAP-S25 plus rifampicin produced 66.7% survival at 7 days versus 0% with PBS/SLAP-S25 alone and one survivor with rifampicin monotherapy; organ bacterial loads were reduced. SLAP-S25-resistant E. coli showed collateral susceptibility to hydrophobic antibiotics.

Abbreviations: ATCC, American Type Culture Collection; BALB/c, Bagg albino laboratory-bred mouse strain; CFU, colony-forming units; CG, clonal group; CR-HvKp/CR-HvKP, carbapenem-resistant hypervirulent Klebsiella pneumoniae; FE-SEM, field-emission scanning electron microscopy; FIC, fractional inhibitory concentration; HepG2, human hepatocellular carcinoma cell line; hvKp/hvKP, hypervirulent Klebsiella pneumoniae; IP, intraperitoneal; ITC, isothermal titration calorimetry; LPS, lipopolysaccharide; MDR, multidrug-resistant; MIC, minimum inhibitory concentration; NPN, 1-N-phenylnaphthylamine; OM, outer membrane; PBS, phosphate-buffered saline; qPCR, quantitative polymerase chain reaction; qRT-PCR, quantitative reverse-transcription polymerase chain reaction; R1-R5, rifampicin-resistant mutant strains; R1'-R5', revertant strains with wild-type rpoB; RFP, rifampicin; RNA, ribonucleic acid; rmpA/rmpA2, regulator of mucoid phenotype A/A2; RpoB, RNA polymerase beta subunit; RpoC, RNA polymerase beta-prime subunit; SD, standard deviation; SLAP-S25, short linear antibacterial peptide S25; ST, sequence type; ZDV, zidovudine.

Supplementary Table S2. Detailed extraction of clinical studies reporting rifampicin-containing therapy in HvKp/HmKp infections

Study	Age/sex	Risk factor(s)	Infection site(s)	Positive sample(s)	Microbiological details	Rifampicin regimen	Other management	Timing/duration	Detailed outcome/notes
Kawaguchi et al., 2023 [24]	70/F	Type 2 diabetes mellitus	Emphysematous pyelonephritis; pyogenic spondylitis	Blood and urine	String-test-positive K. pneumoniae; magA negative and rmpA negative; susceptibility reported in source	Rifampicin 450 mg/day	Meropenem, levofloxacin and later cephalosporin therapy; renal drainage; continuous hemodiafiltration	Started approximately day 20 based on treatment timeline; continued for approximately 50 days	Blood data improved after combination therapy; developed pyogenic spondylitis during course; survived and discharged for rehabilitation
Lin et al., 2021 [25]	29/M	None reported; prior knee fracture	Septic arthritis of right knee, pneumonia and bacteremia	Blood culture; knee effusion culture negative	String-test-positive hypervirulent K. pneumoniae; isolate reported susceptible/pan-sensitive	Rifampicin 0.6 g every 12 h	Meropenem 2 g every 8 h; surgical joint management; several prior antibiotics before final combination	Rifampicin started around day 16; meropenem plus rifampicin given for approximately 10 days	Improved within 3 days of meropenem plus rifampicin; became afebrile and was discharged
Hamada et al., 2024 [26]	82/M	Type 2 diabetes mellitus	Liver abscess, iliopsoas abscess, spondylodiscitis, septic pulmonary embolism and symmetrical peripheral gangrene	Blood cultures and aspirated disc-lavage fluid	String-test-positive hypermucoviscous K. pneumoniae; favourable susceptibility to evaluated antibiotics	Rifampicin 450 mg/day	Cefoperazone/sulbactam plus rifampicin, later meropenem plus rifampicin; drainage/disc lavage and supportive care	Started on day 3 after culture identification and positive string test; exact stop date not clearly specified	Survived; discharged on day 113; antibiotics stopped on day 148 after abscess resolution; toe lesions eventually required amputation
Joshi et al., 2025 [27]	34/M	Splenectomy; uncontrolled diabetes mellitus; diabetic ketoacidosis	Invasive liver abscess syndrome with liver abscess, lung abscesses/cavitations, pneumonia, bacteremia and multisystem involvement	Blood, bronchoalveolar lavage and liver abscess drainage culture	K. pneumoniae isolated; high-mucus phenotype/hypervirulent infection suspected; susceptibility details limited	Rifampin used; dose NR	Meropenem, linezolid, micafungin and later tobramycin plus rifampin; bronchoscopy, liver abscess drainage and intensive care support	Timing and duration NR; rifampin added after clinical deterioration and suspicion of hvKp despite prior antibiotics	Deteriorated after prolonged antibiotics; developed distributive shock and renal failure; died despite aggressive treatment
Shanbhag et al., 2024 [28]	43/M	Uncontrolled type 2 diabetes mellitus	Liver abscess with bacteremia and lung involvement/consolidation with cavity	Blood and liver pus culture	K. pneumoniae isolated from blood and pus; pan-sensitive; string test positive (>5 mm)	Rifampicin 600 mg orally twice daily	Piperacillin/tazobactam initially; changed to meropenem 1 g twice daily and ciprofloxacin 500 mg twice daily; USG-guided pigtail drainage	Rifampicin added on day 3; regimen continued for 4 weeks, followed by rifampicin and ciprofloxacin for 2 additional weeks after discharge	Clinical improvement after 2 days of rifampicin-containing regimen; repeat USG showed no liver collection and chest X-ray improved; discharged
Sokhanvari et al., 2024 [37]	NR	Critical illness/mechanical ventilation context	Ventilator-associated pneumonia due to CR-hvKp	Respiratory culture/sample; details from case report	Carbapenem-resistant hypervirulent K. pneumoniae; exact virulence markers as per source	Rifampin used with colistin; dose NR in simplified extraction	Intravenous and nebulized colistin plus supportive care	Timing/duration NR	Follow-up culture became negative after therapy; survived to discharge
Mitchell et al., 2022 [38]	NR	NR	Suspected disseminated hvKp infection with hepatic, pulmonary, prostate and soft-tissue involvement	Persistent cultures reported in abstract; details limited	Suspected hvKp; conference abstract with limited microbiological detail	Rifampin used with meropenem; dose NR	Meropenem and additional supportive/source-control measures as reported in abstract	Timing/duration NR	Clinical improvement reported; final outcome NR

Abbreviations: CR-hvKp, carbapenem-resistant hypervirulent Klebsiella pneumoniae; F, female; HmKp, hypermucoviscous Klebsiella pneumoniae; HvKp/hvKp, hypervirulent Klebsiella pneumoniae; M, male; NR, not reported; rmpA, regulator of mucoid phenotype A; magA, mucoviscosity-associated gene A; USG, ultrasonography.
